# Supplementary material for: Are carbon emissions trading and green financial instruments synergistic? -Comprehensive quantitative research based on content analysis
Source: PLoS One. 2024 Mar 7;19(3):e0298601. doi: 10.1371/journal.pone.0298601 (PMC10919601; doi:10.1371/journal.pone.0298601)
Supplement: S1 Data — (ZIP) [file pone.0298601.s001.zip › Data/地级市Z_F工作报告.zip!/╣π╬≈/╖└│╟╕█╩╨/└┤╫╘╣1⁄2╓┌║┼í╛┬φ┐╦╩2╛▌═°í┐_2003.docx]

各位代表：

现在，我代表市人民政府向大会报告工作，请予审议，并请市政协各位委员和其他列席人员提出意见。

2002年工作的回顾

过去的一年，我们高举邓小平理论伟大旗帜，全面贯彻“三个代表”重要思想，在市委的正确领导下，在市人大的监督和市政协的支持下，坚持把发展作为第一要务，解放思想，实事求是，与时俱进，开拓创新，克服前进道路上的困难，较好地完成了市二届人大五次会议确定的经济社会发展目标和任务。初步统计，全市完成国内生产总值70.9亿元，增长15%，其中，第一产业增加值20.88 亿元，增长4.5%，第二产业增加值21.63亿元，增长31%，第三产业增加值28.39亿元，增长12.5%。全市经济快速发展，社会进步，政治稳定，民族团结，边疆巩固，人民安居乐业。

港口综合实力明显增强。全市港口货物吞吐量完成1316万吨，增长18.24%，其中防城港1116万吨，增长11.3%，集装箱4.6万标箱，增长1.29倍。港口物流业发展势头强劲，建立了防城港在南宁、贵州都匀两地的物流配送中心，扩大了物流辐射范围，增强了货物疏运能力。港口建设力度加大。11、12号泊位累计完成投资1.9亿元，扩建堆场10万平方米；15万吨级大型船舶减载平台及配套航道项目前期工作加快推进；一批装卸、过驳设施得到更新或改造。国家主枢纽港和西南出海主通道的地位得到进一步巩固和加强。

经济结构调整取得新突破。全市经济结构发生了积极的变化，一、二、三产业占GDP的比重由上年的32.1%、28.4%、39.5%变为29.4%、30.5%、40.1%，产业结构由三、一、二优化为三、二、一，产业层次得到提升。山上、海上特色农业发展较快，农业基础地位进一步加强。全市调整中低产田4.5万亩发展高效农业；新增“三田”4.44万亩；甘蔗品种优质率95%，提高5个百分点；农业生态得到改善，新增水果种植1.23万亩,完成造林19.6万亩，退耕还林1.79万亩;东兴万亩现代农业园、港口区赤沙高科技养殖基地等初具规模，全市新增标准化海水养殖1万亩，水产品总产值14.44亿元，增长4.26%。乡镇企业健康发展，运行质量逐步提高。粮食流通体制、土地流转制度等改革有序推进。工业经济实力逐步壮大。全市工业总产值59.8亿元，增长52.8%。临海工业发展势头良好，引进了“中中外”、“中外外”型等临海加工工业。大海粮油加工一期工程建成投产，实现产值17.2亿元，拉动全市GDP增长4.63个百分点。在加快大海粮油加工二期工程和防城港岳泰饲料厂建设的同时，又成功引进得利塑胶制品厂、台湾森钜工业等一批项目。国有企业放开搞活取得突破性进展，防城港务局、上上糖业有限公司等企业建立现代企业制度步伐加快，完成了防城糖厂的破产收购，全市国有企业改制率60.8%。传统工业的技术更新和改造进一步加快。工业经济效益综合指数139.79%，提高46.79个百分点。旅游、商贸业和民营经济发展较快。全市旅游收入5.46 亿元，增长21.3%。社会消费品零售总额24.29亿元，增长7.2%。个体私营经济创产值3.6亿元，增长4.6%。

项目建设取得新成效。全社会固定资产投资完成16亿元。上思至大寺二级公路（我市段）建成使用，西湾跨海大桥及配套公路累计完成投资1.3亿元，东兴至江山一级公路开工建设。完成东兴“五·七”堤等界河整治工程，维修加固海堤22.5 公里，马正开海堤完成工程量59%，建成三面光渠道30公里，除险加固小峰等水库27 座。东湾临海工业园、东兴工业园规划建设顺利推进。城镇基础设施建设力度加大，市行政中心区开工建设，城市电网改造工程竣工，农村电网二期工程、10万吨水厂和生活垃圾处理厂建设加快，有效推进了城镇化进程。

边境建设大会战取得决定性胜利。按照自治区和市委的部署，把边境建设大会战作为一项“德政工程”、“民心工程”来抓，完成投资2.56亿元，建成了24类1939个边境地区建设项目。边境地区水、电、路、通讯等基础设施得到明显改善，群众行路难、饮水难、住房难、上学难、看病难等问题得到有效解决。与此同时，派出了25个“兴边富民”驻村工作队，深入边境村屯指导农民发展特色经济，完成村集体经济项目48个、基础设施项目135个，促进了村集体经济逐步壮大和农民收入不断提高，在25个边境村中，有21个村的农民人均增收超过200元，个别村达到500元。边境地区经济繁荣、民族和睦、边疆安宁，政府与人民群众的关系进一步密切。

集中整治投资软环境成效显著。全市开展集中整治投资软环境活动，努力转变政府机关作风，推行首问负责制、办事限时制等相关制度，成立投资服务中心、投诉中心、重大项目和企业法制保障中心，规范收费行为，减少审批环节，简化办事程序，提高工作效率，加快口岸通关速度，改善投资环境，促进了对外开放。全市新批准成立外商投资企业11家，实际利用外资2055万美元,利用内资1.45亿元。外贸进出口总额3.36亿美元，增长2.29倍。边贸成交额18.61亿元，增长8.07%。海关税收7.94亿元，增长41%。

财政金融形势良好。大力培植财源税源，加强财税征管，财政收入实现平稳增长。全市财政收入完成5.03亿元，增长11.06%，比年初确定的目标提高3.06个百分点。财税改革力度加大，支出结构进一步优化，保证了干部职工的工资发放，保证了机关的正常运转，保证了重点方面的支出。金融保险体制改革取得新进展，防范和化解风险的能力增强。全年共发放贷款17.07亿元，增长47%，有力支持了地方经济建设。

人民生活进一步改善。城镇居民人均可支配收入7664元，增加1003元；农民人均纯收入2224元，增加198元。扶贫工作力度加大，解决4.2 万人的饮水难问题，全市有1.55万贫困人口跨过温饱线。社会保障体系改革和就业工作取得新成效。安置城镇人员就业4075人，组织跨地区劳务输出3815人，城镇登记失业率控制在3.5%以内。确保了企业离退休人员养老金和失业人员基本生活保障金100%按时足额发放，养老保险社会化发放率达到100%。城镇居民最低生活保障得到落实。

精神文明和民主法制建设继续推进。深入学习党的十五届五中、六中全会和十六大精神，认真实践“三个代表”重要思想，加强爱国主义、社会主义、集体主义和公民道德教育。积极开展“破除迷信、崇尚科学、反对邪教”活动，市民的科学意识进一步提高。“百里边海文明示范带”工程不断推进，“十姐妹学雷锋服务队”学习活动进一步深化。群众性文明创建活动蓬勃开展，文明城市创建工作取得新成效。

坚持向市人大及其常委会报告工作，认真落实人大及其常委会的决议、决定，自觉接受人大及其常委会的监督，积极办理市人大代表议案、建议、批评和意见，全年办复109件，办结率100%。加强与市政协以及各民主党派的联系和沟通，自觉接受人民政协的民主监督，办理政协委员提案261件，办结率100%。法制教育深入开展，“四五”普法全面铺开，市民法律意识进一步增强。各级政府领导坚持深入调查研究，帮助基层和群众解决实际困难和问题，密切了与人民群众的联系。依法行政进一步推进，政府廉政建设得到加强。反腐败工作力度加大，共查办案件94件，挽回经济损失276万元。

社会稳定与安全生产工作力度加大。社会治安综合治理得到加强，“严打”整治斗争成效显著，打击“两抢”、经济犯罪和“黄赌毒”力度加大，与“法轮功”等邪教组织的斗争不断深入。公安队伍在维护社会稳定中发挥了积极的作用。“三大纠纷”专项治理工作取得新成效，有效调处民间纠纷和化解社会矛盾，维护了社会稳定。反走私斗争深入开展，边境口岸管理得到加强。市场经济秩序整治继续推进，市场环境得到进一步改善。安全生产责任制进一步得到落实，防止了重大安全生产事故的发生。

双拥和国防教育工作取得新成绩。全市拥军优属、拥政爱民活动深入开展，通过了全国双拥模范城的检查验收。驻我市人民解放军和武警部队官兵在加强自身建设的同时，积极支援地方经济建设和维护社会稳定，确保了边疆巩固，促进了我市经济与社会发展。国防教育、人民防空、国防后备力量建设成效显著。

各项社会事业全面发展。组织实施了农村科技信息网络、中越星火国际化科技合作示范基地、港口区海洋梯级生态养殖等22个科技项目。“两基”成果得到巩固和发展，国家贫困地区义务教育二期工程稳步实施，中小学危房改造力度加大，建成市高级中学教学楼等教育基础设施，教育水平和质量进一步提高，小学和初中入学率分别达99.8%和94.5%，高考取得建市以来最好成绩。城乡文化日益活跃，文化市场逐步规范，文化基础设施建设得到加强。市中医院门诊楼、市中心血站等卫生项目建成或部分建成，城乡医疗卫生条件不断改善，农村卫生基础设施建设成果显著。群众体育运动广泛开展。计划生育工作取得新成绩，人口自然增长率和人口出生率分别为7.52‰和11.96‰，控制在自治区下达的指标内。国土资源管理力度加大，我市被评为“全国海域使用管理示范区建设工作先进单位”。生态建设与环境保护取得新进展，污染物排放量控制在自治区下达的总量指标内。民政、审计、物价、统计、规划、人事人才、新闻出版、广播电视、口岸查验、工商行政管理、质量技术监督、药品监督管理、防震减灾、气象、档案、修志等各项社会事业在经济建设和社会发展中作出了积极贡献，民族、宗教、外事、侨务、老龄、妇女儿童和残疾人等事业都取得新的成绩。

各位代表，过去一年是近年来我市经济社会发展最快的一年，特别是取得了国民经济快速增长、临海工业与对外贸易两个重大突破和边境建设大会战胜利告捷的新成就。我们深切感到，这些成绩的取得是在自治区党委、自治区人民政府和市委的领导下，深入实践“三个代表”重要思想，以经济建设为中心，把发展作为第一要务，与时俱进，抓住新机遇，开拓新思路，谋求新发展的结果；是贯彻落实市委决策，认真执行市人大及其常委会的决议、决定，广泛吸纳市政协及各民主党派的合理意见和建议,抓住重点，突破难点，培育亮点，加快发展的结果；是防城港驻军、武警部队和中直、区直驻港单位及各界人士大力支持的结果；是全市各族人民团结奋斗、扎实工作的结果。在此，我代表市人民政府向全市广大工人、农民、知识分子，向热情支持、帮助政府工作的人大代表、政协委员、各民主党派、工商联、人民团体和防城港驻军、武警部队及中直、区直驻港单位表示衷心的感谢！对所有为我市改革开放和现代化建设做出贡献的各界人士致以崇高的敬意！

在肯定成绩的同时，我们也清醒地认识到前进中还有不少困难和问题，主要是：农业产业化水平低，工业化、城镇化发展滞后；一些项目建设进展缓慢，投资对经济的拉动不够明显；可用财力有限，支出压力大；铁路“瓶颈”制约突出，港口货物疏运困难，等等。在新的一年里，我们一定要采取有力措施，认真解决存在的各种困难和问题。

2003年工作的主要任务

今年是全面贯彻落实党的十六大精神，加快富民兴市新跨越步伐，全面建设小康社会的重要一年，是实施“十五”计划的关键一年，也是我市建市十周年，做好今年的工作，具有十分重要的意义。

按照市委的总体部署，今年我们要以邓小平理论和“三个代表”重要思想为指导，认真贯彻落实党的十六大精神和中央、自治区经济工作会议精神，按照全面建设小康社会的新要求和市第三次党代会的总体部署，坚持把发展作为执政兴国的第一要务，加快经济结构战略性调整，立足港口发展临海工业，大力推进农业产业化经营，积极培育港口物流、旅游和非公有经济等新的经济增长点；围绕率先实现对外开放新突破，狠抓环境整治、招商引资、扩大出口三个环节，全方位扩大开放，进一步深化改革；继续实施“兴边富民”工程和就业再就业工程，提高城乡居民生活水平和生活质量，维护社会稳定，促进经济跨越式发展和社会全面进步，为全面建设小康社会奠定坚实的基础。

今年全市经济与社会发展的主要预期目标是：国内生产总值增长12%，其中，第一产业增长5%，第二产业增长19%，第三产业增长12%；地方财政收入增长8%；全社会固定资产投资增长15%；全市港口货物吞吐量完成1400万吨，其中防城港1200万吨；外贸进出口总额增长10%；直接利用外资增长12%；社会消费品零售额增长9%；城镇登记失业率控制在3.5%以内；城镇居民人均可支配收入增长8%；农民人均纯收入增长10%；安置城镇就业4500人。

实现上述目标，我们面临许多发展机遇和有利条件：我国加入世贸组织、建立中国——东盟自由贸易区、西部大开发和自治区要求我市率先实现对外开放新突破，为我市加快经济跨越式发展和社会全面进步注入强大的生机与活力；中央继续实施扩大内需的方针、积极的财政政策和稳健的货币政策，有利于我市扩大投资，促进消费，拉动发展；去年我市大力整治投资软环境，有力地促进了对外开放，全市经济呈现了近年来少有的良好发展势头。特别是全市人民通过深入学习党的十六大精神，进一步统一了思想，振奋了精神，充满了奋起直追、加快发展的迫切愿望和强烈要求，这是我们继续前进的强大动力。只要我们坚定不移地以党的十六大精神和“三个代表”重要思想为指导，以全面建设小康社会为目标，以发展为第一要务，充分利用发展机遇和有利条件，聚精会神搞建设，一心一意谋发展，无论遇到多大困难都是可以克服的，实现上述目标是完全可能的，我市的跨越式发展是大有希望的。为此，我们要突出抓好以下事关全局的十项工作：

（一）	加快港口生产与建设，大力发展临海临边工业

加大港口生产与建设力度。按既定的港口功能定位和装卸设备大型化、专业化、现代化的要求，准确把握国际海运发展趋势，积极联合国内外大财团、大企业、大公司共同开发港口资源，大力发展货主码头，实现港口投资主体多元化。加快11、12号泊位的建设，建设10万平方米堆场，逐步完善配套设施，扩大生产能力。推进15万吨级船舶减载平台及配套航道项目建设。继续做好13、14号泊位的前期工作，加强对25万吨、30万吨级超大型泊位和深水航道规划建设的研究。力争引进石化企业建设石化项目配套油码头。加大资金投入，加快设备更新改造。积极开拓货源市场，发挥铁路、公路运输作用，努力提高货物集疏港能力。加大港池回淤治理力度，依法整治西湾海域。

努力壮大临海临边工业。要以港口为依托，实行临海、临边工业并举，走科技含量高、经济效益好、资源消耗低、环境污染少、人力资源优势得到充分发挥的新型工业化道路，努力提高工业总量和质量。要大力发展临海工业。抓住自治区在沿海一带建立外商集中投资区的机遇，做好企沙工业园的前期工作，加快东湾临海工业园的土地平整和道路、供水、供电、通讯等基础设施建设，搞好工业项目招商引资，重点发展粮油、石化、冶金、港口机械、包装材料等产业，力争引进一批与可持续发展战略相配套、符合环境保护要求的临海工业项目。争取大海粮油加工二期工程、森钜PPR板加工、防城港岳泰饲料厂、防城天然香料厂等项目年内投产。要大力发展临边出口加工工业。发挥东兴的区位优势，加快东兴工业园建设，吸引国内外企业投资办厂。重点发展适合东盟市场需求的纺织品、机电产品、绿色食品、保健食品等加工工业。

加快企业的改革与创新。大力推进国有企业产权制度改革和创新，只要不影响经济安全和生态环境，只要有利于壮大工业经济总量，只要有利于提高企业竞争力，都要大力支持，放手发展。要以发展的眼光，鼓励多种经济成份参与国有企业改制。加大港口企业管理创新、服务创新、技术创新力度，提高国际竞争力。对无市场、无效益的企业，要加大兼并、破产力度，推进国有资产合理流动和重组。按照产品创新、降低成本、提高竞争力的要求，继续对制糖、化工、制药、建材和食品加工等企业实施技术改造。要加快企业信息化建设，提高生产和销售的科技含量。

（二）推进农村经济结构调整，促进农民增收农村发展

要继续围绕再造一个“山上防城港”和再造一个“海上防城港”的目标，以建设现代农业为突破口，以市场为导向，以科技创新和体制创新为动力，大力发展特色农业。今年在粮食面积调减的情况下，努力提高单产，使总产量保持与去年持平；甘蔗总产量增长9%；水果总产量增长16%；肉类总产量增长6%；水产品总产量增长2%。

加快农业结构调整和优化。因地制宜，做好“山海”特色农业文章，把特色做优，把规模做大，把优势做强。要发挥现代农业示范园区辐射带动作用,推广先进适用技术，大力实施“种子工程”，提高主要农作物的良种覆盖率。推进高产高效农业，抓好“三田”建设。抓好肉桂、八角品种改良、病虫害防治和先进管护技术的推广，提高单位面积产量和产品质量。推广高产高效安全养殖技术，推进养殖标准化生产；加快发展海水养殖、远海捕捞、海产品加工以及海洋相关产业。加快发展草食动物特别是牛奶业生产。大力发展“无公害”农产品、有机食品、绿色食品。加强以沼气为纽带的生态建设，加强森林资源特别是水源林的保护，推进珠江源头防护林、沿海防护林二期工程建设，年内要完成造林14万亩，速生丰产林4.5万亩，退耕还林5.5万亩。加强农业基础设施建设，重点抓好病险水库的除险加固，加快马正开等海堤标准化建设，完成长歧干渠和上思渠道33公里硬化工程，开工建设防城区咤管坡护岸等中越界河整治项目，确保农业灌溉和安全度汛。

努力提高农业产业化经营水平。加快农业领域的对外开放，吸引外商和民营企业到我市投资创办农业和农产品加工企业。把发展乡镇企业同推进农业产业化建设结合起来，采用“企业+基地+农户”等模式，加快乡镇企业小区建设，培植一批辐射面广、带动力强的“龙头企业”，发展水产品、香料、金花茶等特色农副产品加工和出口创汇农业，增加农产品附加值，增强市场竞争力。加快农产品质量安全检验检测体系和认证体系建设，加强农产品的质量安全监督管理。强化农业营销网络建设，重点抓好海产品、香料等大宗农产品的流通工作。推进农村税费改革和粮食流通体制改革，积极探索农村土地流转机制，促进农业生产要素合理流动和优化配置。

加大扶贫开发力度。继续做好贫困地区农民增收项目的组织实施，从一、三产业中优选一批覆盖面大、效益好的农民增收项目，逐项分解到乡、村、屯，努力实现村村屯屯都有增收项目，确保农民增收。进一步搞好以贫困村为重点的农村基础设施和生态建设。扎实开展定点帮扶和社会扶贫，不断扩大技术扶贫、智力扶贫、生态扶贫。要坚决查处加重农民负担的各种违法违纪行为，切实减轻农民负担。

深入实施“兴边富民”工程。各级政府要把改善人民生活放在更加突出的位置，以富民的目标凝聚人心，以富民的举措鼓舞人心，以富民的实绩安定人心。特别是驻村工作队要做到目标不变、队伍不散、力度不减，深入边境地区、边远山区指导农民发展经济林果业、农产品加工、边境贸易、边境旅游、边境运输等特色经济，努力壮大村集体经济实力。加大边境地区和边远山区的投入，进一步完善水、电、路等基础设施，提高农民的科技文化素质，促进边境地区和边远山区经济发展和边民致富。大力培育城乡劳动力市场，组织农民外出务工，促进农村富余劳动力的有序转移。鼓励和支持外出务工人员返乡创业，加快农村经济发展。

（三）加大项目建设力度，努力推进城镇化进程

大力推进项目建设。今年全社会固定资产投资要力争达到25亿元。要进一步优化投资布局，调整投资结构，抓住投资重点，集中力量推进关系我市经济发展全局的重大项目建设，努力实现“三个十”：一是年内建成西湾跨海大桥、大海粮油二期工程、防城港岳泰饲料厂、市10万吨水厂、防城港11—12号泊位、市农村电网二期工程，续建东兴至江山一级公路、疏港大道、防城港物流园区、市生活垃圾处理厂等十大项目；二是新开工市行政中心区基础设施、市中心区行政办公用房及配套设施、东湾临海工业园基础设施、防城港15万吨级减载平台及配套航道、防城港5万吨级航道改造、上思平福水电厂、东兴国际展览中心一期工程、东兴市边境工业园基础设施、东兴市城区护岸工程、东兴市水厂扩建等十大项目。三是做好企沙工业园区规划及基础设施、防城港13、14号泊位、东海湾航道、滨海公路防城港至龙门段、市污水处理厂、防城区防洪排涝工程、江山半岛景区旅游度假区基础设施、中心区三甲医院、东兴中越二桥、沿海捕捞渔民转业转产等十大项目的前期工作。为此，要深化投融资体制改革，大力筹措资金，争取国债、财政专项资金和银行信贷资金支持；通过市场途径，大力培育多元投资主体，激活社会投资，引导民间资本参与项目建设。要强化投资和项目建设目标管理责任制，加强对重点项目、国债项目专项稽查和检查，规范管理，落实责任，把投资和项目管理纳入程序化、规范化、法制化轨道。

全力推进市行政中心区开发建设。要把市行政中心区的开发建设作为今年城镇化建设的重中之重，尽快拉开城市主体框架。上半年动工建设市中心区行政办公用房主体工程，加快建设中心区环路工程、滨海大道、东西大道、南北大道等城市路网，逐步配套水、电、通讯等城市公用设施。积极鼓励和支持市内外单位、部门和个人到中心区投资建设，加快房地产开发，推动住房消费。要用市场经济的理念经营城市，推进城镇建设投资主体多元化，形成政府引导、市场运作、全社会参与开发的格局。

加快小城镇建设。要以企沙镇、江平镇、那良镇、峒中镇、在妙镇等为重点，搞好城镇道路、供电、给排水等设施建设，发挥城镇在区域经济特别是促进农村经济发展中的辐射带动作用。落实加快推进城镇化进程的各项政策措施，逐步解决户籍、土地、社保等问题，消除不利于城镇化发展的体制和政策障碍。结合创建文明城市，继续实施绿化、美化、亮化、净化工程，建设西湾东海岸等城市景观，提高城市品位。

（四）加快发展现代物流和旅游业，培育新的经济增长点

大力发展现代物流业。以构建服务大西南与东南亚的区域性物流中心为目标，以市场为导向，大力发展中转贸易和加工贸易，完善防城港在南宁、贵州都匀两地的物流中心，加快防城港食糖配送中心建设，逐步形成有色金属矿、非金属矿以及粮油、饲料批发等分销配送中心；积极探索连锁经营、物流配送、网上购物等现代流通方式，扩大物流辐射范围，以物流带动人流、资金流、信息流、项目流，拉动市场消费。进一步实施“大通关”工程，抓好信息网络平台、交通运输网络、口岸基础设施、仓储配套设施建设，加快通关速度。要把物流业和商贸业结合起来，推进国有商业企业改革，加速商品与要素市场体系的培育。

提升旅游业发展水平。继续打造“上山下海出国”特色旅游品牌，加大旅游项目的招商引资力度，多元化筹措资金，争取西部国债，重点推进江山半岛旅游度假区、十万大山国家森林公园、金滩旅游度假区等景区景点及配套设施建设。开通防城港至越南下龙湾海上航线。抓好沿海港口、京族风情、沿边公路旅游线、金花茶观赏园等市内旅游。完成旅游信息大厦的装修并投入使用。特别要以我市建市十周年为契机，加大旅游宣传促销力度，提高知名度，吸引更多游客。加大旅游市场整治力度，理顺旅游管理体制，规范市场秩序，优化旅游环境。加快培养现代旅游管理和导游人才，提高服务水平。

促进新兴服务业健康发展。适应城镇化发展的需要，加快发展教育培训、文化娱乐、卫生保健、体育健身等服务业。推进电子政务和电子商务建设，提高全市信息化水平。进一步发展会计、监理、咨询、法律等中介服务业。积极发展房地产和物业管理，开发住房二级市场，扩大住房消费。鼓励个人借贷消费，培育新的消费热点。

（五）不断改善发展环境，率先实现对外开放新突破

加强治理投资软环境的长效管理。各级政府要推广去年投资软环境集中整治的经验，围绕提高机关效能和率先实现对外开放的新突破，开展一次思想大解放的再教育和大讨论，一切妨碍发展的思想观念都要坚决冲破，一切束缚发展的做法和规定都要坚决改变，一切影响发展的体制弊端都要坚决革除，从制度、机制、政策以及人文环境上解决不适应投资需要的问题，规范各种收费行为，建立开放式的办证大厅，减少审批环节，简化办事程序，进一步提高工作效率。要将“治软”的好办法制度化，建立和完善政务公开、行政事务处置限时、行政效能目标管理等制度，从根本上建立投资软环境的长效管理机制。要用好、用足各项优惠政策，落实优质服务的各项承诺，增强公开性和透明度。依法保护投资者和个体私营经济从业人员的合法权益，通过营造良好的政策环境、服务环境、市场环境、法制环境和人文环境，使我市成为创业成本低、市场秩序好、社会信用高、人居环境优美安全的城市。

实现招商引资新突破。中国——东盟自由贸易区的建立，将为我市提供更广阔的市场，更丰富的资源和跨国优化资源配置的机会。我们要抓住这一机遇，更好地发挥大通道的作用，加快构建“一区三岛”（东兴市区，江山半岛、渔万岛、企沙半岛）和沿边境线一带的对外开放新格局，积极争取国家把我市列入中国——东盟自由贸易区试点区域。要落实招商引资责任制，健全和完善激励机制，调动干部群众参与招商的积极性。采取走出去、请进来的方法,开展灵活多样、切实有效的招商引资活动，发挥侨乡优势，吸引更多的国内外企业到我市投资开发。要完善招商项目库，选择一批有特色、符合国内外市场需求、投资回报率高、对客商有吸引力的重大优势项目进行重点招商。鼓励和引导外资参与国有企业改组、改造，参与企业技术开发和技术创新。继续搞好横向经济联合，发挥驻外“窗口”的作用，加强与友好城市的联系与合作，积极接纳东部地区的产业转移，寻求与港澳台地区更多的经济合作。

扩大对外贸易。实行多元化的外贸发展战略，以国有企业、外资企业、民营企业为主体，重组外贸企业优势；实行一般贸易、加工贸易、边境贸易等多种贸易方式并举，积极扩大对东盟的出口、经济技术和劳务合作，抓好芒街中国商业城等项目建设，带动设备出口和劳务输出。调整出口产品结构，巩固和扩大食用油、桉木片等优势产品出口，加大高新技术产品和机电产品出口比重，提高技术含量和附加值。抓好出口生产基地建设，大力发展加工贸易。推进东兴口岸二期工程，加快峒中、里火等互市点基础设施建设，规范边贸经营秩序，促进边境贸易健康发展。

（六）加大财源建设力度，切实做好金融工作

夯实财源基础。要把培植财源与经济结构调整和发展外向型经济结合起来，大力建设稳固财源，提高经济增长的质量和效益。培育农产品加工龙头企业，提高农产品附加值，巩固和强化农业基础财源;加快临海临边工业园区建设，发展仓储、加工工业，壮大工业支柱财源;大力发展现代物流和特色旅游业，培育第三产业新兴财源;加大出口商品基地建设，扩大开放型财源;积极盘活城镇土地和存量资产，支持和引导民营经济发展，培育新的财源增长点。

加强财政管理。要落实“加强征管，堵塞漏洞，惩治腐败，清缴欠税”的工作方针，依法强化税收征管，实行目标责任制，严厉打击偷、逃、骗税行为，做到应收尽收。进一步调整和优化财政支出结构，保政权运转，保工资发放，保法定支出。对上级下拨的专项资金，要确保专款专用；对部门的专项支出，要量财办事，统筹兼顾。要勤俭办一切事业，压缩一般性支出，坚决刹住铺张浪费之风，坚决把会议费、招待费、差旅费等支出中不合理的部分压下来。严格执行预算，加强预算外资金管理和审计监督，整顿和规范财经秩序。推进部门预算和国库集中收付制度改革，着力构建公共财政。

搞好金融保险工作。各级政府及政府各部门要积极支持金融工作，努力建立良好的银企、银贸关系，协调、支持金融部门整顿金融秩序，盘活不良资产，确保金融业的稳健经营。金融部门要继续执行中央稳健的货币政策，推进金融体制改革，创新金融产品，强化金融监管，优化信贷结构，防范金融风险。要围绕我市经济建设和投资重点，加大信贷资金支持力度。继续拓展保险业务，努力提高服务质量。

（七）大力发展民营经济，努力抓好就业和再就业工作

加快民营经济发展。要充分发挥民营经济在促进经济增长、扩大就业和活跃市场等方面的重要作用。要创造良好环境，降低门坎，放宽市场准入条件，放手发展民营经济，在投融资、土地使用、税收、信贷、对外贸易等方面给予民营企业与国有企业同等待遇，实现公平竞争。要破除一切阻碍民营经济发展的体制弊端，毫不动摇地鼓励、支持和引导个体、私营等非公有制经济投资一、二、三产业，发展劳动密集型产业，特别是满足群众衣食住行各方面需要的服务业。鼓励和引导民营企业参与国有企业改组改造，推动各种所有制经济在产权上联合，在机制上对接，在经营方式上取长补短。要选择基础条件好、区位优势突出的区域，规划建设民营企业创业园，在人才培训、信息指导等方面给予大力支持，加强相关服务，努力培育一批民营名牌企业、名牌产品。对为我市改革开放作出突出贡献的个体户、私营企业要给予宣传表彰，营造政府推动、典型引路、市场聚集、舆论鼓舞、环境吸引的良好氛围。

拓宽就业和再就业渠道。要认真贯彻执行中央、自治区关于扶持就业和再就业的政策措施，落实各项扶持和优惠政策，加大对下岗失业人员等弱势群体的帮扶工作力度，引导群众转变择业观念，鼓励自谋职业和自主创业。加强就业技能培训和劳动力市场建设，完善就业服务体系，抓好大中专毕业生就业和城镇退役军人安置工作，帮助更多的人员实现就业和再就业。

加快社会保障体系改革。完善养老、失业和城镇居民最低生活保障制度，切实把“两个确保”和“一个低保”落到实处。进一步完善医疗保险制度。认真做好救灾救济工作，加大对农村特困户、灾区群众和残疾人的帮扶力度，落实优抚安置政策，切实保障社会特殊群体和特困家庭的基本生活。

（八）深入实施可持续发展战略，保持经济社会协调发展

优先发展科技和教育。要以“科技金源”、“科技金桥”、“科技金穗”三大科技行动为载体，全面实施新一轮的科技创新计划。大力引进先进适用技术，培植我市的技术创新能力，重点引进和推广农业新技术、新品种，加快工业产品更新换代，增强市场竞争力。优化教育资源配置，发展素质教育、职业教育和民族教育，推进市高级中学和电大分校建设。积极推进事业单位人事制度、分配制度改革。大力实施人才兴市战略，加大人才培训力度，完善选人用人和激励机制，营造良好的创业环境，进一步调动各类人才积极性。

加强人口、环境和资源保护工作。要完善人口与计划生育目标管理责任制，全市人口自然增长率控制在10.27‰以内。加大环境管理和监测力度，加强海洋、河流环境综合整治，建立环保公众参与机制，全面推进生态示范市建设。做好国土资源规划和管理，尽快建立有形土地市场，确保各类资源合理开发和永续利用。

全面发展各项社会事业。加强文化基础设施建设，加快市公共图书馆、边境民族艺术馆等项目的建设，推进“知识工程”、“百里边海文明示范带”建设。深化文化体制改革，鼓励社会力量兴办文化产业，促进文化市场健康发展。实施广播电视网络升级改造工程，加快广播电视网络的整合。要深化城镇医疗卫生体制改革，强化卫生监督执法。重视农村卫生工作，加强重大疾病的预防控制和妇幼保健工作。实施全民健身计划，发展竞技体育。工商行政管理、质量技术监督、药品监督管理、物价、统计、气象、测绘、档案、修志、防震减灾等各项事业都要有新的发展。

（九）坚持三个文明一起抓，切实维护社会稳定

加强精神文明建设。各级政府和政府各部门要在集中精力加强物质文明建设的同时，加强精神文明建设，深入学习党的十六大精神，全面贯彻“三个代表”重要思想，坚持党的基本理论、基本路线和基本纲领，巩固和加强马克思主义的指导地位，弘扬爱国主义、集体主义、社会主义精神，为经济建设提供精神动力。加强国民教育、社会文化、休闲娱乐等各种思想文化阵地的建设和管理。加强公民道德建设，提升社会公德、职业道德和家庭美德水平。普及科学知识，反对封建迷信，倡导文明健康的生活方式。大力开展文明城市、文明村镇、文明社区、文明行业等群众性精神文明创建活动，全面提高市民素质和城市文明程度。

推进民主法制建设。大力建设政治文明，加强依法治市，提高民主化、法制化水平。各级政府要自觉接受同级人民代表大会及其常委会的监督，主动听取人民政协的意见。认真办好人大代表议案、建议、批评和意见，认真办好政协委员提案。发挥工会、共青团、妇联等群众团体在民主管理、民主监督、维护群众权益方面的作用。加强同民主党派合作共事，做好外事、侨务、宗教、民族和对台工作，巩固和发展广泛的爱国统一战线。加强基层政权和群众性自治组织建设，进一步完善政务、厂务、村务公开制度，坚持领导接待日制度，建立健全民主决策、民主管理、民主监督的程序和制度。加强“四五”普法工作，增强市民法律意识。帮扶法律弱势群体，做好法律援助工作。

继续整顿和规范市场经济秩序。要大力提倡诚实守信的市场道德，逐步建立覆盖企业和个人的信用档案，规范经营行为。继续整顿和规范建筑市场、房地产市场和文化市场，严厉打击制售假冒伪劣商品、走私贩私、偷税漏税、传销和变相传销等违法犯罪行为。整顿和规范市场价格秩序，加大食品、药品、农资等关系人民群众切身利益和安全的商品市场秩序的整治力度。

抓好社会稳定与安全生产工作。继续深入开展“严打”整治斗争，依法严惩各种刑事犯罪、经济犯罪和黑恶势力，坚决扫除“黄赌毒”等社会丑恶现象，强化对“法轮功”重点人员的监控。全面落实社会治安综合治理各项措施，加大“三大纠纷”调处力度，努力解决开发区遗留问题，预防和妥善处理群体性事件。严格执行《安全生产法》，加强安全生产监督管理。继续以矿山、危险化学品、易燃易爆物品和道路、水上交通为重点，开展安全生产专项整治，突出抓好重点行业、重点地区和重点企业安全事故隐患的监控，防止重大安全事故发生。

努力建设双拥模范城。搞好军政、军民团结，支持军队现代化建设，充分发挥部队在经济建设和社会发展中的积极作用，切实加强人民防空、民兵和预备役工作。深入开展拥军优属、拥政爱民活动。提高全市人民的国防意识，树立国门的良好形象。加强边疆建设，维护边境稳定。

（十）转变政府职能，加强政风建设

不断夯实理论基础。在知识经济和信息化迅猛发展的新的历史时期，全体政府工作人员必须适应新形势、新变化，把学习放在更加重要的位置，加强马克思主义、毛泽东思想、邓小平理论和“三个代表”重要思想的学习，运用马克思主义的立场、观点和方法，解决工作中的实际问题；认真学习科技、财政、金融、对外贸易、法律法规等方面的知识，进一步增强驾驭市场经济的能力。

加快政府职能转变。按照社会主义市场经济的要求，推进政企分开、政事分开，使政府职能真正转到宏观调控、市场监督、社会管理和公共服务上来。要进一步加快行政审批制度改革，优化发展环境。加快全市政府系统政务信息网络建设，提高行政效率。要全面推行政务公开，对政府决策、行政管理、公共事务等重大事项，都要依法向社会公开，自觉接受人民群众的监督。

弘扬艰苦奋斗精神。各级政府和政府各部门要贯彻胡锦涛总书记在西柏坡学习考察时的讲话精神，以党中央提出的“八个坚持，八个反对”为准则，大力倡导艰苦创业、奋发图强、勤俭办事、求真务实的优良传统和作风，坚决克服官僚主义、形式主义，深入实际调查研究，倾听群众呼声，关心群众疾苦，解决人民群众反映强烈和不满意的问题，提高政府机关的服务水平。

加大依法行政力度。全体政府工作人员要增强依法行政意识，学会运用法律手段管理行政、经济、文化与社会事务。要自觉维护法律法规的权威，确保政令畅通。建立健全行政效能监察监督制度。强化行政复议、行政诉讼对行政执法的监督作用，推动政府管理行为的规范化、法制化。政府各级各部门的领导干部要带头依法行政，真正做到有权必有责、用权受监督、侵权要赔偿。要加强执法队伍建设，培养一支高素质的行政执法队伍。

加强政府廉政建设。反腐倡廉，教育是基础，法制是保证，监督是关键。要加强廉洁自律，加强对政府工作人员的教育和管理，认真执行廉洁从政行为准则，正派做人，公道办事。认真落实廉政建设责任制，健全和完善各项规章制度，实行领导干部述职述廉制度，推行民主评议制度和领导干部经济责任审计制度。加强对干部特别是领导干部的监督，从源头上防止和治理腐败。对那些违法乱纪、以权谋私、贪污腐化的人，必须彻底查处，树立人民政府的良好形象。

各位代表！

党的十六大描绘了全面建设小康社会的宏伟蓝图，市委确定了实现富民兴市新跨越的发展目标，为我们进一步指明了前进方向。新的形势催人奋进，新的事业任重道远。让我们紧密团结在以胡锦涛同志为总书记的党中央周围，以邓小平理论和“三个代表”重要思想为指导，紧紧依靠全市各族人民，解放思想，实事求是，与时俱进，开拓创新,为实现我市经济社会跨越式发展，全面建设小康社会而努力奋斗！
